# INTESTINAL PERFORATION CAUSED BY COVID-19

**DOI:** 10.1590/0102-672020190001e1515

**Published:** 2020-11-20

**Authors:** Sergio Carlos NAHAS, José Donizeti de MEIRA-JÚNIOR, Lucas Faraco SOBRADO, Maurício SORBELLO, Vanderlei SEGATELLI, Edson ABDALA, Ulysses RIBEIRO-JÚNIOR, Ivan CECCONELLO

**Affiliations:** 1 Gastrointestinal and Colorectal Surgery Divisions, Department of Gastroenterology, Hospital das Clínicas, School of Medicine, University of São Paulo, Cancer Institute of the State of São Paulo, São Paulo, SP, Brazil; 2 Department of Pathology, School of Medicine, University of São Paulo, Cancer Institute of the State of São Paulo, São Paulo, SP, Brazil; 3 Department of Infectious and Parasitic Diseases, School of Medicine, University of São Paulo, Cancer Institute of the State of São Paulo, São Paulo, SP, Brazil

**Keywords:** SARS-CoV-2, Colorectal cancer, Acute abdomen, Thrombosis, Colorectal surgery, SARS-CoV-2, Câncer colorretal, Abdome agudo, Trombose, Coloproctologia

## INTRODUCTION

As the coronavirus disease 2019 (COVID-19) pandemic spreads throughout the world, new clinical manifestations are being reported. In addition to the respiratory manifestations, acute renal failure^3^, hypercoagulability^9^, vomiting and diarrhea^5^ have been described. 

The Cancer Institute of the São Paulo State (ICESP) has already performed over 8500 surgeries for colorectal cancer in the last 10 years. It is one of the hospitals associated with the University of São Paulo School of Medicine, which has already admitted over 3000 patients with moderate or severe COVID-19 for in-hospital treatment. We present a case of intestinal perforation caused by microcirculatory thrombosis in the colon in a patient undergoing surgery for colorectal cancer.

## CASE REPORT

A 92-year-old male patient with a diagnosis of rectal adenocarcinoma sought emergency care in April 2020 due to intestinal subocclusion. He had a personal history of hypertension and nondialysis chronic kidney disease. Chest and abdomen CT scans showed no pulmonary changes; multiple liver metastases, the largest one measuring 3.0 cm and distension of the colon and small intestine. He underwent exploratory laparotomy, and a tumor was found in the upper rectum, causing bowel obstruction. A rectosigmoidectomy was performed with blind-ending closure of the rectal stump and terminal colostomy.

**FIGURE 1:**
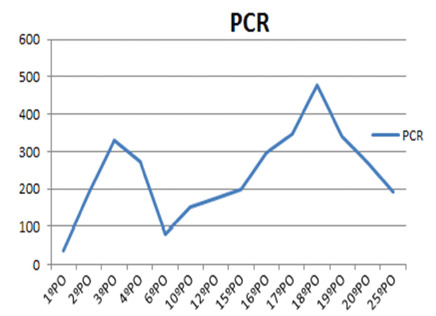
Serum CRP curve

During the postoperative (PO) period, the patient received food and had intestinal transit until the 3rd PO day, when he started to present coughing and fever. Laboratory testing showed increased C-reactive protein (CRP), as shown in Figure 1. Chest CT scan revealed consolidation in the right lung base. Assessment by the infectious disease team indicated a clinical and radiological profile compatible with bacterial pneumonia. Antibiotic therapy with piperacillin-tazobactam was initiated and maintained for five days with good response, after which the patient started receiving levofloxacin. He was discharged on the 8th PO day, with clinical improvement, decreased CRP levels, good acceptance of food and a functioning colostomy.

After two days, on the 10th PO day, he returned to the emergency room complaining of diffuse abdominal pain, oliguria and coughing. Abdominal examination showed a nonfunctioning colostomy and abdominal pain upon palpation, without signs of peritonitis. In the laboratory analysis, the patient had leukocytosis of 19,000 cells/mm³, with 92% neutrophils and a CRP level of 110 mg/l (normal range <5.0 mg/l) in addition to renal dysfunction with an increase in creatinine levels from 1.58 mg/dl to 3.7 mg/dl and an increase in urea from 56 mg/dl to 110 mg/dl. A CT scan of the abdomen showed pneumoperitoneum without free fluid collections and with diffuse distension of small bowel loops. Exploratory laparotomy was indicated and showed punctiform perforation of the descending colon at 5 cm from the colostomy, with fecal peritonitis blocked by small bowel loops. The perforated descending colon segment located at 5 cm from the colostomy was resected, followed by exhaustive washing of the cavity, terminal colostomy and introduction of antibiotic therapy with meropenem.

**FIGURE 2:**
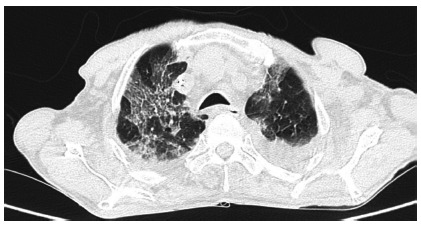
Chest CT scan taken on the 5th PO day

Due to the concomitant pulmonary manifestations, the patient was referred to the ICU intubated with vasoactive drugs, a nasogastric tube, antibiotic therapy and parenteral nutrition. He maintained high nasogastric tube output. Starting on the 1st PO day, he received anticoagulant therapy for the prophylaxis of thromboembolic events. He was extubated on the 3rd PO day and discharged from the ICU. On the 5th PO day, worsening of the respiratory condition was observed, with discomfort, decreased oxygen saturation and increased CRP. The chest CT scan, Figure 2, showed multiple bilateral ground-glass opacities. A D-dimer level of 3225 ng/ml and DHL of 638 U/l were observed. A nasopharyngeal and oropharyngeal swab was collected to screen for severe acute respiratory syndrome coronavirus 2 (SARS-CoV-2). Due to the decrease in oxygen saturation even with 100% oxygen supplementation by mask, the patient was again admitted to the ICU, immediately subjected to orotracheal intubation, and kept on mechanical ventilation and in isolation.

Regarding the clinical evolution, the swab was positive, with worsening of general conditions: adynamic ileus, acute renal failure with creatinine reaching 5.0 mg/dl, need for high doses of vasoactive drugs and antibiotic therapy with meropenem, vancomycin and anidulafungin. The patient was extubated on the 28th PO day after improvement of the respiratory condition. He was discharged from the ICU on the 30th PO day but progressed to a coma vigil, according to the neurologist. Death occurred on the 36th PO day.

**FIGURE 3:**
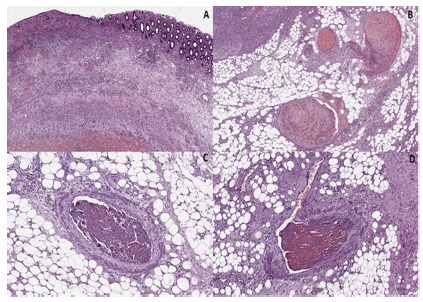
Microscopic findings: A) partial or total necrosis of the intestinal mucosa with edema, inflammation and areas of hemorrhage (4x, H&E); B, C and D) areas of necrosis of adipose tissue and vascular changes, such as congestion and organized thrombi (20x, H&E).

An anatomopathological assessment of the surgical specimen revealed thrombotic changes in the microcirculation of the perforated descending colon (Figure 3).

## DISCUSSION

We report the case of a patient who underwent emergency surgical treatment for colorectal cancer without signs or symptoms of COVID-19. At the time of admission, there was no suspicion of COVID-19, and a nasopharyngeal and oropharyngeal swab was not collected for RT-PCR screening for the virus. However, during the PO evolution of the disease, when the patient began to show signs and symptoms compatible with COVID-19, testing was performed, and SARS-CoV-2 infection was confirmed; the infection led to renal, pulmonary and gastrointestinal complications, which led to the his death. The patient’s severe respiratory symptoms were reversed with intensive support and multidisciplinary treatment; the most significant factor in the unfavorable outcome of the case was the involvement of the digestive tract, with intestinal perforation, abdominal sepsis and multiple organ dysfunction secondary to the infectious process.

Gastrointestinal symptoms such as diarrhea, nausea and vomiting have been described in COVID-19 patients; they can occur even in the absence of respiratory symptoms^5^ and are related to intestinal inflammation^2^ caused by the viral process.

To the best of our knowledge, this is the first report in the literature of intestinal perforation resulting from microcirculation thrombosis associated with SARS-CoV-2 infection with histopathological evaluation.

In addition to respiratory and gastrointestinal symptoms, SARS-CoV-2 infection produces an inflammatory response triggered by rapid viral replication and cell destruction, resulting in the recruitment of macrophages and monocytes and inducing the release of cytokines and chemokines such as IL-1β, IL-2, IL-6, IL-7 and IFN-γ^5^. These cytokines induce the formation of thrombin through the release of tissue factors by endothelial cells and the disruption of natural anticoagulant mechanisms. In addition, fibrinolysis is impaired by the increase in plasminogen activator inhibitor 1, which impairs fibrin clearance^6^ ^-^ ^10^. Thus, this cytokine storm^6^ eventually induces activation of the coagulation cascade, causing thrombotic phenomena, which may manifest in various vascular territories.

Arteriolar thrombi have been described in the pulmonary microcirculation^1^ in both areas of normal lung parenchyma and areas of tissue damaged by the effects of the virus. A study^7^ by our Department of Pathology demonstrated the benefit of anticoagulant therapy for improving the oxygenation of critical patients, and this procedure was applied in the reported case. We found in the literature a report of acute intestinal necrosis^4^ secondary to the microcirculatory thrombotic effects of the new coronavirus. In addition to pulmonary and intestinal thrombosis, there was a significant increase in the incidence of deep vein thrombosis in ICU patients due to COVID-19; the incidence reached 85%, even when prophylactic anticoagulants were used8. Skin lesions have been described in patients with a severe form of COVID-19 as a result of thrombotic disorders^11^.

The arteriolar thrombi visualized in this case and represented in Figure 3 are similar to the thrombi in the pulmonary circulation described in autopsies^1^ of patients who died from COVID-19.

The macroscopic study of the surgical sample showed a segment of the colon measuring 4.5 cm in length with serosa containing fibrinous material. The intestinal wall was thin and had a focal perforation area. The mucosa was hemorrhagic. In the microscopic findings, we observed changes in the ischemic pattern with partial or total necrosis of the intestinal wall and areas of mucous loss, edema, inflammation and hemorrhage. In the subserosa, necrosis of the adipose tissue with congestion and several organized vascular thrombi (Figure 3). Most likely, the intestinal microcirculatory thrombosis observed in the presented case led to ischemia of the colon wall, culminating in intestinal perforation, which caused the need for surgical re-exploration.

 Considering the observation of the potential complications caused by COVID-19 in the PO period, the hospital infection control committee began collecting swabs to screen for SARS-COV-2 in all patients before emergency surgical procedures. For elective surgeries, tests are performed 48 h before the procedure, and if positive, surgery is postponed whenever possible.
